# Refill adherence and persistence to lipid‐lowering medicines in patients with type 2 diabetes: A nation‐wide register‐based study

**DOI:** 10.1002/pds.4281

**Published:** 2017-08-11

**Authors:** Sofia Axia Karlsson, Christel Hero, Björn Eliasson, Stefan Franzén, Ann‐Marie Svensson, Mervete Miftaraj, Soffia Gudbjörnsdottir, Katarina Eeg‐Olofsson, Karolina Andersson Sundell

**Affiliations:** ^1^ Department of Public Health and Community Medicine, Sahlgrenska Academy University of Gothenburg Sweden; ^2^ Department of Molecular and Clinical Medicine, Sahlgrenska Academy University of Gothenburg Sweden; ^3^ National Diabetes Register, Centre of Registers Gothenburg Sweden; ^4^ AstraZeneca AB, Medical Evidence and Observational Research Gothenburg Sweden

**Keywords:** lipid‐lowering medicines, maximum gap method, medication possession ratio, persistence, refill adherence, type 2 diabetes

## Abstract

**Purpose:**

This study aimed to describe and compare refill adherence and persistence to lipid‐lowering medicines in patients with type 2 diabetes by previous cardiovascular disease (CVD).

**Methods:**

We followed 97 595 patients (58% men; 23% with previous CVD) who were 18 years of age or older when initiating lipid‐lowering medicines in 2007–2010 until first fill of multi‐dose dispensed medicines, death, or 3 years. Using personal identity numbers, we linked individuals' data from the Swedish Prescribed Drug Register, the Swedish National Diabetes Register, the National Patient Register, the Cause of Death Register, and the Longitudinal Integration Database for Health Insurance and Labour Market Studies. We assessed refill adherence using the medication possession ratio (MPR) and the maximum gap method, and measured persistence from initiation to discontinuation of treatment or until 3 years after initiation. We analyzed differences in refill adherence and persistence by previous CVD in multiple regression models, adjusted for socioeconomic status, concurrent medicines, and clinical characteristics.

**Results:**

The mean age of the study population was 64 years, 80% were born in Sweden, and 56% filled prescriptions for diabetes medicines. Mean MPR was 71%, 39% were adherent according to the maximum gap method, and mean persistence was 758 days. Patients with previous CVD showed higher MPR (3%) and lower risk for discontinuing treatment (12%) compared with patients without previous CVD (P < 0.0001).

**Conclusions:**

Patients with previous CVD were more likely to be adherent to treatment and had lower risk for discontinuation compared with patients without previous CVD.

## 1 INTRODUCTION

Adults with diabetes have increased risk for cardiovascular disease (CVD) and mortality compared with adults without diabetes.^1^, ^2^, ^3^ Such risk often associates with comorbidities and lifestyle factors (eg, hypertension, dyslipidemia, obesity, physical inactivity, and smoking), particularly in patients with type 2 diabetes. Additionally, previous CVD increases the risk for recurrent CVD events.^4^ Therefore, therapeutic guidelines for diabetes care recommend antihypertensive and lipid‐lowering medicines in addition to glucose‐lowering treatment.^5^

Adherence and persistent treatment are essential to obtaining a treatment effect.^6^ Adherence is the extent to which a person follows agreed recommendations from a prescriber. Persistence represents the duration of time from initiation to discontinuation of treatment.^7^, ^8^ Different methods of measuring adherence provide similar values.^9^, ^10^ Compared with other adherence measures, register data yield reliable estimates, particularly regarding pharmacy claims (ie, refill adherence).^8^, ^10^ Currently, adherence and persistent treatment are far from optimal, especially in chronic conditions,^8^ posing a risk for insufficient treatment effect and increasing risk for morbidity and mortality.

Although refill adherence to lipid‐lowering medicines in the general population varies between studies, it is often higher among patients with diabetes and/or previous CVD.^11^, ^12^, ^13^, ^14^ Few studies have assessed persistence to lipid‐lowering medicines for longer than 2 years 11, 15 or estimated refill adherence and persistence to lipid‐lowering medicines in patients with type 2 diabetes only. The present study aimed to assess and compare refill adherence and persistence to lipid‐lowering medication in monotherapy among patients with type 2 diabetes by previous CVD during an observation period of 3 years.

**KEY POINTS**

- The overall refill adherence during the study period was 71% measured with MPR among 97 595 patients with type 2 diabetes included in the study; 39% had no gaps exceeding 45 days.
- Average persistence was 758 days in the total population.
- Patients with type 2 diabetes and previous CVD had a higher refill adherence measured with MPR and were less likely to have gaps in treatment compared with patients with type 2 diabetes and no previous CVD.
- Patients with type 2 diabetes and previous CVD were more persistent to treatment compared with patients with type 2 diabetes and no previous CVD.

## 2 METHODS

### 2.1 Study population

In the Swedish Prescribed Drug Register (SPDR), we identified patients aged ≥18 years and registered in the National Diabetes Register (NDR) with type 2 diabetes, who initiated use of lipid‐lowering medicines between 1 January 2007 and 31 December 2010 (the index period). Our study distinguished between NDR‐registered patients with type 1 and type 2 diabetes by applying the epidemiological definition of type 2 diabetes. Such individuals receive treatment with diet and/or other glucose‐lowering medicines than insulin, or experience onset of diabetes at age ≥40 years and receive insulin treatment and/or other glucose‐lowering medicines.^16^, ^17^, ^18^, ^19^, ^20^

**Figure 1:**
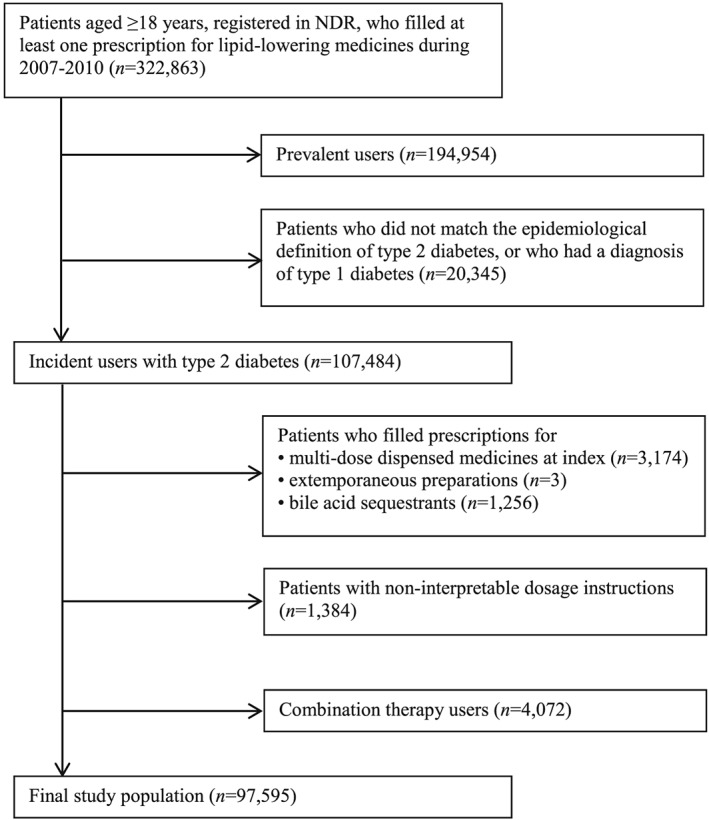
Exclusion criteria for the study population

To identify incident users of lipid‐lowering medicines, we established a washout period encompassing the 366 days preceding the first day of filled prescription (the index date). Our study excluded patients who (1) filled either extemporaneously prepared prescriptions for lipid‐lowering medicines that lacked information about package size, or bile acid sequestrants more frequently prescribed for indications other than hyperlipidemia;^21^ or (2) used a combination of different lipid‐lowering substances or different strengths of the same substance (Figure 1). Combination therapy comprised prescriptions for (1) more than 1 substance or multiple strengths of the same substance filled on the same date, or (2) a previously filled substance/strength that was filled again within 45 days after finishing the previous supply of that substance/strength and filling another substance/strength during the gap. Multiple lipid‐lowering substances in the same unit (eg, tablet) were considered monotherapy. We followed all patients until the first fill date of multi‐dose dispensed medicines (because these were automatically dispensed even if the patient never redeemed the medicines), death or 3 years after the index date, whichever occurred first.

### 2.2 Data sources

Patients' unique Swedish personal identity number allowed us to link information from the SPDR, the NDR, the National Patient Register, and the Cause of Death Register (all administered by the Swedish National Board of Health and Welfare) as well as the Longitudinal Integration Database for Health Insurance and Labour Market Studies (LISA) (managed by Statistics Sweden). The regional ethics review board approved our study (No. 563‐12).

Filled prescriptions were collected from the SPDR, which individualizes its data on all prescriptions filled since 1 July 2005,^22^ including information about age, sex, type of medicine, package size, date of dispensing, and free text dosage instructions from the prescriber. We gathered clinical data from the NDR, which has maintained nation‐wide records on diabetes care in Sweden since 1996.^23^ We obtained individual data on socioeconomic status from the LISA database^24^ and received data on CVD and cancer diagnoses from The National Patient Register.^25^ The Cause of Death Register provided data on date of death.^26^

### 2.3 Estimation of days' supply and overall observation period

We used SPDR data to estimate the number of days with medicines on hand and the overall observation period. To determine the duration of each prescription, we divided the number of filled units (eg, tablets) by the interpreted daily dosage, based on the free text variable. We considered dosage instructions interpretable if they stated number of doses per day (eg, 1 tablet/day). Our study excluded patients with non‐interpretable dosage instructions such as variable (eg, 1–2 tablets) or non‐existent dosage information (eg, as prescribed). Furthermore, we developed an algorithm to interpret the free text variable and validated it on a predefined random sample (5% of the dosage instructions), requiring at least 95% concordance. More than 98% of the reviewed dosage instructions matched the daily dosage assigned by the algorithm.

We added overlapping supply between 2 identical prescriptions to the most recent prescription. If substance or strength differed between 2 prescriptions, we canceled the prescription on the day before patients started the new substance/strength and removed any remaining supply.

### 2.4 Refill adherence and persistence

We based our adherence estimates on the medication possession ratio (MPR)^9^, ^10^ and the maximum gap method.^27^, ^28^, ^29^ To calculate MPR, which represents the proportion of days with medicines on hand during the observation period, we divided the total days' supply by the total number of observation days. In this study, MPR is a continuous variable. To compare our results with studies that categorize MPR exceeding 80% as adherent behavior, we divided MPR into quintiles.^11^

The maximum gap method identifies gaps between filled prescriptions, allowing patients to be without medicines on hand for a predefined time period without defining them as non‐adherent.^11^ We defined a gap as ≥45 days between 2 prescriptions. Thus, patients with no gaps were adherent. We based the cutoff for gap length on the Swedish reimbursement system, which allows patients to fill a maximum of 3‐month's supply per refill,^30^ the most common practice for lipid‐lowering medicines. We also estimated the mean number of gaps and mean number of days within gaps for non‐adherent patients.

We defined persistence as the duration between initiation and discontinuation of treatment^9^, ^11^ and discontinuation as a gap of ≥180 days between 2 filled prescriptions (representing 2 refills within the reimbursement scheme). The discontinuation date was the last day with medicines on hand before the first discontinuation gap. We estimated persistence from the index date to the discontinuation date or the end of the observation period, whichever occurred first. To estimate the annual discontinuation rate, we divided the number of patients who discontinued treatment during each year by the total number of patients who were persistent at the start of each year.

### 2.5 Sensitivity analyses

Using the maximum gap method, we estimated the stability of the 45‐day cutoff by assessing 2 alternative gap lengths (30 and 90 days). Furthermore, Censored patients did not have the same possibility to fulfill the 180‐day discontinuation gap cutoff; thus, to estimate the impact of immortal time bias, we assessed alternative lengths of the discontinuation gap cutoff (90 and 135 days) in patients who were censored during the observation period.

### 2.6 Covariates

Potential confounders of refill adherence and discontinuation of treatment included (1) socioeconomic status (age, sex, country of birth, marital status, level of education, employment status, profession, and individual disposable income); (2) concurrent medicines (diabetes medicines, anticoagulants, and antihypertensive medicines); and (3) clinical characteristics (diabetes duration, glycated hemoglobin [HbA1c], blood‐lipid levels, blood pressure, estimated glomerular filtration rate [eGFR], microalbuminuria, macroalbuminuria, cancer diagnosis, body mass index [BMI], physical activity, and smoking).

We collected socioeconomic covariates on the index date ± 12 months. Country of birth included Sweden, other Nordic countries, other EU27 countries,^31^ rest of Europe/the Soviet Union, Africa, the Americas, or Asia/Oceania. Marital status encompassed unmarried, married/registered partner, divorced, or widow/widower. Level of education included compulsory school or lower, upper secondary school, or post‐secondary. Categories for employment status comprised unemployed, employed, or retired (≥65 years of age and registered as unemployed). Individual disposable income is shown as Swedish Krona (SEK) and categorized in quartiles. We categorized profession as upper white collar, lower white collar, blue collar, or other.

We collected patients' prescriptions for concurrent medicines for 18 months prior to the index date. Categories for diabetes medicines included no diabetes medicines, insulin only, other glucose‐lowering medicines only (including both oral and injectable glucose‐lowering medicines), or a combination of insulin and other glucose‐lowering medicines. Anticoagulants were categorized as no anticoagulants, antiplatelets (excluding heparins), or other anticoagulants. Antihypertensive medicines comprised no antihypertensive medicines, angiotensin‐converting‐enzyme (ACE) inhibitors/angiotensin‐II‐receptor blockers (ARBs), beta blockers, calcium channel antagonists, diuretics, or other antihypertensive medicines. Each patient might have filled prescriptions for more than 1 anticoagulant and/or antihypertensive medicine.

Data are often reported to NDR in retrospect; thus, data on blood‐lipid levels were collected between 24 months before and 14 days after the index date. Data on other clinical characteristics were collected between 24 months before and 12 months after the index date, choosing the value closest to index. We included cancer diagnoses that occurred up to 5 years before the index date. HbA1c, blood‐lipid levels, and blood pressure were categorized according to recommended target values^32^ existing at the time of the study. BMI and eGFR were categorized according to recommended references values.^33^, ^34^ We dichotomously categorized microalbuminuria, macroalbuminuria, cancer diagnosis, and smoking (at least 1 cigarette/pipe per day or stopped smoking within 3 months). Physical activity was defined as a 30‐minute walk or equivalent and categorized as < once per week, 1–2 times/week, 3–5 times/week, or daily.

Previous CVD included any ischemic heart disease, atrial fibrillation, heart failure, cerebrovascular disease, peripheral vascular disease, coronary artery graft bypass, percutaneous coronary intervention, and/or leg amputation occurring between 1997 and the index date. For ICD‐10 and operation codes, see Supplementary Appendix 1.

### 2.7 Statistical analyses

We analyzed differences in refill adherence and persistence according to previous CVD (no previous CVD considered the reference) in 3 multivariable regression models based on the potential confounders' character. The first model adjusted for socioeconomic status and the second model adjusted for socioeconomic status and concurrent medicines. The third (fully adjusted) model included socioeconomic status, concurrent medicines, and clinical characteristics. The reference categories are marked “ref” in Table 3.

We used multiple linear regression to analyze differences in MPR, and Cox proportional hazard regression and Kaplan‐Meier to analyze differences in discontinuation. Difference in gap occurrence were analyzed with logistic regression adjusted for all potential confounders in the fully adjusted model.

Data management and statistical analyses were performed using SAS Software Version 9.4 (SAS Institute, Cary NC).

## 3 RESULTS

### 3.1 Study population

Table 1 shows the characteristics of the study population (97 595 patients; 57.8% men). Of these, 22.7% had a history of CVD. The mean age was nearly 64 years, and the average diabetes duration was >5 years. Approximately 80% of patients were born in Sweden, 55.2% were married/registered partner, and 47.9% were employed. Twenty percent filled insulin prescriptions, and 44.4% filled no prescriptions for diabetes medicines. Around 30% filled prescriptions for anticoagulants, and 59.7% filled prescriptions for antihypertensive medicines. Mean HbA1c was 54.2 mmol/mol (7.1%), and average LDL‐cholesterol was 3.5 mmol/L. Approximately 25% were physically active less than once a week, 44.2% had a BMI ≥30, and 16.9% were smokers.

**Table 1:** Baseline characteristics for the study population (*n* = 97 595)

		Total population *(n =* 97 595)		Previous CVD *(n =* 22 131)		No previous CVD *(n =* 75 464)	
Variables		*n*	%	*n*	%	*n*	%
Sex	Men	56 396	57.8	14 465	65.4	41 931	55.6
Age	18‐40	2391	2.5	93	0.4	2298	3.1
(years)	41‐60	33 641	34.5	4143	18.7	29 498	39.1
	61‐80	55 356	56.7	14 509	65.6	40 847	54.1
	>80	6207	6.4	3386	15.3	2821	3.7
	Mean (SD)	63.8	(11.3)	69.6	(10.4)	62.1	(11.0)
	Median	64.0	‐	70.0	‐	63.0	‐
Country of birth	Sweden	77 857	79.8	18 039	81.5	59 818	79.3
	Other Nordic country	5531	5.7	1432	6.5	4099	5.4
	Other EU27 country	3235	3.3	814	3.7	2421	3.2
	Rest of Europe/the Soviet Union	4013	4.1	871	3.9	3142	4.2
	Africa	1205	1.2	147	0.7	1058	1.4
	The Americas	884	0.9	138	0.6	746	1.0
	Asia/Oceania	4855	5.0	687	3.1	4168	5.5
Marital status	Unmarried	15 100	15.6	2577	11.8	12 523	16.7
	Married/registered partner	53 539	55.2	11 533	53.0	42 006	55.9
	Divorced	17 804	18.4	4151	19.1	13 653	18.2
	Widow/widower	10 508	10.8	3508	16.1	7000	9.3
Level of education	Compulsory school or lower	36 853	38.9	10 027	47.2	26 826	36.5
	Upper secondary school	41 384	43.6	8314	39.1	33 070	44.9
	Post‐secondary	16 617	17.5	2918	13.7	13 699	18.6
Employment status	Employed	46 460	47.9	6932	31.8	39 528	52.6
	Unemployed	13 731	14.2	2500	11.5	11 231	14.9
	Retired^a^	36 760	37.9	12 337	56.7	24 423	32.5
Profession	Upper white collar	23 242	31.4	4418	30.3	18 824	31.7
	Lower white collar	7481	10.1	1408	9.6	6073	10.2
	Blue collar	40 593	54.9	8027	55.0	32 566	54.9
	Others	2651	3.6	749	5.1	1902	3.2
Individual	1^st^ quartile	24 219	25.0	6190	28.4	18 029	24.0
disposable	2^nd^ quartile	24 240	25.0	6960	32.0	17 280	23.0
income (SEK)	3^rd^ quartile	24 237	25.0	4913	22.6	19 324	25.7
	4^th^ quartile	24 255	25.0	3706	17.0	20 549	27.3
	Mean (SD)	190 080	(404 772)	173 978	(512 230)	194 742	(367 716)
	Median	152 600	‐	137 500	‐	159 200	‐
Diabetes medicines	No diabetes medicines	43 322	44.4	10 392	47.0	32 930	43.6
	Insulin only	9399	9.6	2662	12.0	6737	8.9
	Other glucose‐lowering medicines only	34 990	35.9	6543	29.6	28 447	37.7
	Insulin and other glucose‐lowering medicines	9884	10.1	2534	11.5	7350	9.7
Anticoagulants^b^	No anticoagulants	67 501	69.2	9066	41.0	58 435	77.4
	Antiplatelets (excl. heparins)	25 523	26.2	10 350	46.8	15 173	20.1
	Other anticoagulants	5773	5.9	3577	16.2	2196	2.9
Antihypertensive	No antihypertensive medicines	39 301	40.3	6699	30.3	32 602	43.2
medicines^b^	ACE inhibitor/ARBs	44 684	45.8	11 622	52.5	33 062	43.8
	Beta blockers	3553	3.7	1265	5.7	2288	3.0
	Calcium channel antagonists	20 552	21.1	5580	25.2	14 972	19.8
	Diuretics	33 050	33.9	10 024	45.3	23 026	30.5
	Other antihypertensive medicines	1577	1.6	589	2.7	988	1.3
Diabetes duration	Mean (SD)	5.4	(7.1)	6.6	(8.1)	5.1	(6.7)
(years)	Median	3.0	‐	4.0	‐	3.0	‐
HbA1c	<42 [<5]	9766	14.5	1932	13.9	7834	14.6
(mmol/mol [%])	42‐52 [5‐6]	28 231	41.8	5699	40.9	22 532	42.0
	>52 [>6]	29 562	43.8	6297	45.2	23 265	43.4
	Mean (SD)	54.2 [7.1]	(14.0 [3.4])	54.3 [7.1]	(13.4 [3.4])	54.2 [7.1]	(14.1 [3.4])
	Median	51.0 [6.8]	‐	51.0 [6.8]	‐	51.0 [6.8]	‐
Total cholesterol	<4.5	4772	11.1	1524	19.0	3248	9.3
(mmol/L)	≥4.5	38 288	88.9	6483	81.0	31 805	90.7
	Mean (SD)	5.6	(1.0)	5.3	(1.1)	5.7	(1.0)
	Median	5.6	‐	5.3	‐	5.6	‐
LDL‐cholesterol	<2.5	4882	12.8	1364	19.7	3518	11.2
(mmol/L)	≥2.5	33 359	87.2	5554	80.3	27 805	88.8
	Mean (SD)	3.5	(0.9)	3.2	(0.9)	3.5	(0.9)
	Median	3.4	‐	3.2	‐	3.5	‐
HDL‐cholesterol	<1.0 (men) or <1.3 (women)	13 376	33.9	2530	35.5	10 846	33.5
(mmol/L)	≥1.0 (men) or ≥1.3 (women)	26 097	66.1	4588	64.5	21 509	66.5
	Mean (SD) Men/Women	1.2/1.4	(0.4)/(0.4)	1.2/1.3	(0.4)/(0.4)	1.2/1.4	(0.4)/(0.4)
	Median Men/Women	1.1/1.3	‐	1.1/1.3	‐	1.1/1.3	‐
Triglycerides	<2.0	24 846	62.5	4536	63.1	20 310	62.4
(mmol/L)	≥2.0	14 907	37.5	2653	36.9	12 254	37.6
	Mean (SD)	2.0	(1.3)	1.9	(1.2)	2.0	(1.3)
	Median	1.7	‐	1.7	‐	1.7	‐
eGFR	<60	8838	13.8	3425	26.1	5413	10.6
(mL/min/1.73 m^2^)	≥60	55 164	86.2	9705	73.9	45 459	89.4
	Mean (SD)	83.6	(23.8)	75.2	(24.7)	85.7	(23.0)
	Median	82.3	‐	74.3	‐	84.1	‐
BMI	<18.5	161	0.3	44	0.3	117	0.2
(kg/m^2^)	18.5‐24.9	10 042	15.6	2308	17.8	7734	15.1
	25.0‐29.9	25 649	39.9	5351	41.3	20 298	39.6
	≥30.0	28 413	44.2	5269	40.6	23 144	45.1
	Mean (SD)	30.0	(5.3)	29.5	(5.3)	30.1	(5.3)
	Median	29.3	‐	28.8	‐	29.4	‐
Systolic pressure	<130	18 031	26.9	3906	28.3	14 125	26.6
(mmHg)	≥130	48 985	73.1	9915	71.7	39 070	73.5
	Mean (SD)	138.0	(17.0)	137.9	(18.5)	138.0	(16.7)
	Median	136.0	‐	138.0	‐	136.0	‐
Diastolic pressure	<80	29 062	43.4	7254	52.5	21 808	41.0
(mmHg)	≥80	37 954	56.6	6567	47.5	31 387	59.0
	Mean (SD)	78.5	(9.9)	76.2	(10.3)	79.1	(9.7)
	Median	80.0	‐	77.0	‐	80.0	‐
Microalbuminuria	Yes	8922	17.0	2312	23.1	6610	15.6
	No	43 517	83.0	7679	76.9	35 838	84.4
Macroalbuminuria	Yes	3822	6.7	1254	10.9	2568	5.6
	No	53 462	93.3	10 270	89.1	43 192	94.4
Other diseases	Cancer diagnosis	4095	4.2	1430	6.5	2665	3.5
Physical activity^c^	< once per week	13 856	24.9	3842	33.9	10 014	22.6
	1‐2 times/week	11 500	20.7	2237	19.8	9263	20.9
	3‐5 times/week	12 696	22.8	2103	18.6	10 593	23.9
	Daily	17 549	31.6	3139	27.7	14 410	32.5
Smoker^d^	Yes	10 671	16.9	1794	13.9	8877	17.7
	No	52 489	83.1	11 086	86.1	41 403	82.3

a If aged ≥65 years and unemployed.

b Each patient may have filled prescriptions for more than 1 substance within this category.

c 30‐min walk or equivalent.

d At least 1 cigarette or pipe per day or stopped smoking within 3 months.

In patients with previous CVD, 65.4% were men (mean age = 70 years) compared with 55.6% in patients without previous CVD (mean age = 62 years). Patients with previous CVD were retired and had received a cancer diagnosis to greater extent, as well as were more likely to use anticoagulants and antihypertensive medicines than patients without previous CVD.

### 3.2 Refill adherence

Mean MPR in the total study population was 70.9% (Table 2). 76.3% for patients with previous CVD, and 69.3% for patients without previous CVD. Adjusted for potential confounders, the difference in MPR for previous CVD was 2.9%–6.3% (*P* < 0.0001) (Table 3), suggesting greater refill adherence to lipid‐lowering treatment than patients without previous CVD. Country of birth accounted for largest difference in MPR. In the fully adjusted model, MPR for patients born in another European country, Africa, or the Americas was lower (3.3%–3.9%, 12.2%, 11.8% lower, respectively) than patients born in Sweden (*P* < 0.0001). Moreover, MPR was higher (3.8%–4.4%) in patients who filled prescriptions for other glucose‐lowering medicines than patients who filled no prescriptions for diabetes medicines (*P* < 0.0001). The maximum gap method revealed that 61.1% of the total study population had at least 1 gap (mean number of days within a gap = 275). Patients without previous CVD were categorized as non‐adherent more frequently than patients with previous CVD (*P*<0.0001).

**Table 2:** Refill adherence and persistence to lipid‐lowering medicines in patients with type 2 diabetes by previous CVD

		Total population *(n =* 97 595)		Previous CVD *(n =* 22 131)		No previous CVD (*n* = 75 464)	
		*n*	%	*n*	%	*n*	%
MPR (%)	0‐20	11 810	12.1	2017	9.1	9793	13.0
	21‐40	9775	10.0	1803	8.2	7972	10.6
	41‐60	9111	9.3	1741	7.9	7370	9.8
	61‐80	13 054	13.4	2495	11.3	10 559	14.0
	81‐100	53 845	55.2	14 075	63.6	39 770	52.7
	Mean (SD)	70.9	(31.1)	76.3	(29.5)	69.3	(31.4)
	Median	84.7	‐	91.2	‐	82.1	‐
Gaps ≥45 d	Non‐adherent patients^a^	59 656	61.1	11 419	51.6	48 237	63.9
	Number of gaps						
	Mean (SD)	1.7	(0.9)	1.6	(0.9)	1.7	(0.9)
	Median	1.0	‐	1.0	‐	1.0	‐
	Number of days within gaps						
	Mean (SD)	274.7	(285.5)	272.3	(281.9)	275.2	(286.3)
	Median	140.0	‐	138.0	‐	140.0	‐
Persistence	One year	70 742	72.5	16 310	73.7	54 432	72.1
	Two years	59 664	61.1	13 498	61.0	46 166	61.2
	Three years	54 954	56.3	12 123	54.8	42 831	56.8
	Mean (SD) days	758.0	(419.9)	761.4	(411.8)	757.0	(422.2)
	Median days	1095	‐	1095	‐	1095	‐
Discontinuation^b^	Single prescription filled	7365	7.6	1869	8.5	5496	7.3
	Within the first year	24344	25.0	4202	19.0	20142	26.7
	During the second year	9422	13.3	1950	12.0	7472	13.7
	During the third year	3221	5.4	655	4.9	2566	5.6

a Statistically significant difference between patients with and without previous CVD (*P* < 0.0001).

b Annual discontinuation rate is based on number of persistent patients at the start of each year.

**Table 3:** Differences in MPR and hazard ratios for discontinuation of treatment by previous CVD adjusted for potential confounders

		Model 1 (*n* = 73 816)				Model 2 (*n* = 73 816)				Model 3 (*n* = 19 966)			
		MPR		Discontinuation		MPR		Discontinuation		MPR		Discontinuation	
		Estimate (95% CI)	*P*‐value	Hazard Ratio (95% CI)	*P*‐value	Estimate (95% CI)	*P*‐value	Hazard Ratio (95% CI)	*P*‐value	Estimate (95% CI)	*P*‐value	Hazard Ratio (95% CI)	*P*‐value
Previous CVD	No	ref	ref	ref	ref	ref	ref	ref	ref	ref	ref	ref	ref
	Yes	5.9 (5.3, 6.5)	<0.0001	0.76 (0.74, 0.79)	<0.0001	6.3 (5.8, 6.9)	<0.0001	0.75 (0.72, 0.78)	<0.0001	2.9 (1.6, 4.2)	<0.0001	0.91 (0.85, 0.98)	0.0110
Sex	Women	ref	ref	ref	ref	ref	ref	ref	ref	ref	ref	ref	ref
	Men	0.7 (0.2, 1.2)	0.0043	0.97 (0.94, 0.99)	0.0096	1.0 (0.5, 1.5)	0.0001	0.95 (0.93, 0.98)	0.0005	1.9 (0.9, 2.9)	0.0002	0.90 (0.86, 0.95)	0.0001
Age (continuous)	Years	0.3 (0.2, 0.3)	<0.0001	0.99 (0.99, 0.99)	<0.0001	0.2 (0.2, 0.3)	<0.0001	0.99 (0.99, 0.99)	<0.0001	0.1 (0.1, 0.2)	<0.0001	1.00 (0.99, 1.00)	0.0043
Country of birth	Sweden	ref	ref	ref	ref	ref	ref	ref	ref	ref	ref	ref	ref
	Other Nordic countries	−3.1 (−4.1,−2.1)	<0.0001	1.16 (1.10, 1.22)	<0.0001	−3.3 (−4.3, −2.3)	<0.0001	1.17 (1.11, 1.23)	<0.0001	−3.3 (−5.3, −1.2)	0.0017	1.16 (1.05, 1.28)	0.0042
	Other EU27 countries	−5.2 (−6.6, −3.9)	<0.0001	1.30 (1.21, 1.39)	<0.0001	−5.3 (−6.6, −3.9)	<0.0001	1.31 (1.22, 1.40)	<0.0001	−3.9 (−6.8, −1.0)	0.0089	1.19 (1.03, 1.38)	0.0158
	Rest of Europe/the Soviet union	−5.0 (−6.4, −3.6)	<0.0001	1.28 (1.19, 1.38)	<0.0001	−5.0 (−6.4, −3.6)	<0.0001	1.29 (1.20, 1.38)	<0.0001	−3.5 (−6.8, −0.2)	0.0354	1.25 (1.07, 1.47)	0.0050
	Africa	−12.8 (−15.2, −10.5)	<0.0001	1.74 (1.57, 1.92)	<0.0001	−12.6 (−14.9, −10.2)	<0.0001	1.72 (1.55, 1.90)	<0.0001	−12.2 (−17.1, −7.3)	<0.0001	1.64 (1.33, 2.02)	<0.0001
	The Americas	−12.3 (−14.7, −9.9)	<0.0001	1.71 (1.54, 1.90)	<0.0001	−12.0 (−14.4, −9.6)	<0.0001	1.68 (1.51, 1.87)	<0.0001	−11.8 (−17.3, −6.4)	<0.0001	1.81 (1.44, 2.27)	<0.0001
	Asia/Oceania	−2.8 (−4.2, −1.4)	<0.0001	1.17 (1.09, 1.25)	<0.0001	−2.6 (−4.0, −1.2)	0.0002	1.16 (1.09, 1.25)	<0.0001	−1.6 (−4.7, 1.4)	0.2910	1.10 (0.94, 1.28)	0.2336
Marital status	Married/Registered partner	ref	ref	ref	ref	ref	ref	ref	ref	ref	ref	ref	ref
	Unmarried	−1.4 (− 2.0, −0.7)	<0.0001	1.06 (1.02, 1.09)	0.0022	−1.4 (− 2.0, 0.7)	<0.0001	1.06 (1.02, 1.09)	0.0024	−1.3 (− 2.5, 0.0)	0.0519	1.04 (0.97, 1.11)	0.2911
	Divorced	−4.2 (−4.8, −3.6)	<0.0001	1.22 (1.18, 1.26)	<0.0001	−4.1 (−4.7, −3.5)	<0.0001	1.21 (1.18, 1.25)	<0.0001	−4.0 (−5.2, −2.8)	<0.0001	1.20 (1.13, 1.27)	<0.0001
	Widow/Widower	−2.0 (−2.9, −1.2)	<0.0001	1.10 (1.05, 1.15)	0.0001	−2.0 (−2.9, −1.1)	<0.0001	1.10 (1.05, 1.15)	<0.0001	−2.0 (−3.6, 0.3)	0.0198	1.05 (0.96, 1.15)	0.2645
Level of education	Compulsory school	ref	ref	ref	ref	ref	ref	ref	ref	ref	ref	ref	ref
	Secondary school	−1.3 (−1.8, −0.8)	<0.0001	1.06 (1.03, 1.09)	<0.0001	−1.3 (−1.8, −0.8)	<0.0001	1.06 (1.03, 1.09)	<0.0001	−1.3 (−2.3, −0.3)	0.0107	1.06 (1.00, 1.11)	0.0412
	Post‐secondary	−2.1 (−2.8, −1.3)	<0.0001	1.11 (1.07, 1.15)	<0.0001	−1.9 (−2.6, −1.2)	<0.0001	1.10 (1.06, 1.15)	<0.0001	−2.1 (−3.6, −0.7)	0.0045	1.10 (1.02, 1.19)	0.0144
Employment status	Unemployed	ref	ref	ref	ref	ref	ref	ref	ref	ref	ref	ref	ref
	Employed	−1.1 (−1.9, −0.2)	0.0180	1.03 (0.99, 1.08)	0.1674	−1.0 (−1.9, −0.2)	0.0200	1.03 (0.99, 1.08)	0.1813	−0.4 (−2.2, 1.4)	0.6485	1.04 (0.95, 1.14)	0.3674
	Retired^a^	−2.1 (−3.1, −1.1)	<0.0001	1.11 (1.06, 1.17)	<0.0001	−1.9 (−2.8, −0.9)	0.0002	1.10 (1.04, 1.15)	0.0004	−0.8 (−2.7, 1.2)	0.4407	1.06 (0.96, 1.17)	0.2540
Profession	Blue collar	ref	ref	ref	ref	ref	ref	ref	ref	ref	ref	ref	ref
	Upper white collar	−0.1 (−0.7, 0.5)	0.7147	1.00 (0.97, 1.04)	0.9219	−0.1 (−0.7, 0.5)	0.7787	1.00 (0.97, 1.03)	0.9761	−0.1 (−1.3, 1.1)	0.8671	1.03 (0.97, 1.10)	0.3160
	Lower white collar	−0.1 (−0.9, 0.6)	0.7223	1.01 (0.97, 1.05)	0.7380	−0.1 (−0.9, 0.7)	0.8040	1.01 (0.97, 1.05)	0.7782	0.0 (−1.6, 1.5)	0.9489	1.01 (0.94, 1.10)	0.7547
	Other	−0.3 (−1.5, 1.0)	0.6721	1.02 (0.95, 1.09)	0.6491	−0.1 (−1.3, 1.2)	0.9257	1.01 (0.94, 1.08)	0.8650	0.3 (−2.0, 2.6)	0.7948	0.99 (0.87, 1.12)	0.8777
Individual disposable income	1st quartile	ref	ref	ref	ref	ref	ref	ref	ref	ref	ref	ref	ref
	2nd quartile	1.3 (0.6, 2.0)	0.0004	0.92 (0.89, 0.96)	<0.0001	1.3 (0.6, 2.1)	0.0002	0.92 (0.89, 0.96)	<0.0001	2.3 (0.9, 3.7)	0.0011	0.86 (0.80, 0.93)	<0.0001
	3rd quartile	2.5 (1.8, 3.3)	<0.0001	0.87 (0.84, 0.91)	<0.0001	2.5 (1.7, 3.2)	<0.0001	0.88 (0.84, 0.91)	<0.0001	2.2 (0.8, 3.7)	0.0023	0.87 (0.81, 0.93)	0.0002
	4th quartile	3.3 (2.5, 4.1)	<0.0001	0.85 (0.81, 0.89)	<0.0001	3.2 (2.3, 4.0)	<0.0001	0.85 (0.82, 0.89)	<0.0001	3.0 (1.4, 4.6)	0.0002	0.84 (0.77, 0.91)	<0.0001
Diabetes medicines	No diabetes medicines	‐	‐	‐	‐	ref	ref	ref	ref	ref	ref	ref	ref
	Insulin only	‐	‐	‐	‐	−5.5 (−6.3, −4.7)	<0.0001	1.30 (1.25, 1.36)	<0.0001	1.2 (−0.5, 2.8)	0.1674	0.94 (0.87, 1.03)	0.1782
	Other glucose‐lowering medicines only	‐	‐	‐	‐	−0.7 (−1.2, −0.2)	0.0064	1.05 (1.02, 1.08)	0.0002	4.4 (3.3, 5.5)	<0.0001	0.84 (0.79, 0.89)	<0.0001
	Insulin and other glucose‐lowering medicines	‐	‐	‐	‐	−1.4 (−2.1, −0.6)	0.0006	1.09 (1.05, 1.14)	<0.0001	3.8 (2.3, 5.4)	<0.0001	0.85 (0.78, 0.92)	<0.0001
Anticoagulants	No	‐	‐	‐	‐	ref	ref	ref	ref	ref	ref	ref	ref
	Yes	‐	‐	‐	‐	−1.6 (−2.2, −1.1)	<0.0001	1.08 (1.05, 1.11)	<0.0001	0.2 (−0.8, 1.3)	0.6317	0.97 (0.92, 1.02)	0.2632
Antihypertensive medicines	No	‐	‐	‐	‐	ref	ref	ref	ref	ref	ref	ref	ref
	Yes	‐	‐	‐	‐	2.9 (2.4, 3.3)	<0.0001	0.88 (0.86, 0.91)	<0.0001	3.4 (2.5, 4.4)	<0.0001	0.88 (0.84, 0.92)	<0.0001
Diabetes duration	Years (continuous)	‐	‐	‐	‐	‐	‐	‐	‐	−0.2 (−0.3, −0.2)	<0.0001	1.01 (1.01, 1.02)	<0.0001
Total cholesterol (mmol/L)	≥4.5	‐	‐	‐	‐	‐	‐	‐	‐	ref	ref	ref	ref
	<4.5	‐	‐	‐	‐	‐	‐	‐	‐	3.0 (1.1, 4.9)	0.0016	0.88 (0.79, 0.97)	0.0097
LDL‐cholesterol (mmol/L)	≥2.5	‐	‐	‐	‐	‐	‐	‐	‐	ref	ref	ref	ref
	<2.5 mmol/L	‐	‐	‐	‐	‐	‐	‐	‐	−0.5 (−2.2, 1.2)	0.5568	1.02 (0.93, 1.11)	0.7198
HDL‐cholesterol (mmol/L)	≥1.0 (men)/≥1.3 (women)	‐	‐	‐	‐	‐	‐	‐	‐	ref	ref	ref	ref
	<1.0 (men)/<1.3 (women)	‐	‐	‐	‐	‐	‐	‐	‐	1.1 (0.1, 2.2)	0.0293	0.94 (0.89, 0.99)	0.0299
Triglycerides (mmol/L)	≥2.0	‐	‐	‐	‐	‐	‐	‐	‐	ref	ref	ref	ref
	<2.0	‐	‐	‐	‐	‐	‐	‐	‐	0.7 (−0.3, 1.7)	0.1492	0.96 (0.91, 1.01)	0.0859
HbA1c (mmol/mol [%])	>52 [6]	‐	‐	‐	‐	‐	‐	‐	‐	ref	ref	ref	ref
	<42 [5]	‐	‐	‐	‐	‐	‐	‐	‐	1.7 (0.3, 3.2)	0.0185	0.93 (0.86, 1.00)	0.0478
	42‐52 [5‐6]	‐	‐	‐	‐	‐	‐	‐	‐	1.7 (0.7, 2.7)	0.0007	0.92 (0.87, 0.97)	0.0010
eGFR/mL/min/1.73^c^	≥60	‐	‐	‐	‐	‐	‐	‐	‐	ref	ref	ref	ref
	<60	‐	‐	‐	‐	‐	‐	‐	‐	−0.1 (−1.6, 1.4)	0.8866	1.02 (0.94, 1.10)	0.7045
BMI (kg/m^2^)	≥30	‐	‐	‐	‐	‐	‐	‐	‐	ref	ref	ref	ref
	<30	‐	‐	‐	‐	‐	‐	‐	‐	−0.2 (−1.1, 0.8)	0.6979	1.00 (0.95, 1.05)	0.9676
Systolic blood pressure (mmHg)	≥130	‐	‐	‐	‐	‐	‐	‐	‐	ref	ref	ref	ref
	<130	‐	‐	‐	‐	‐	‐	‐	‐	−0.4 (−1.4, 0.7)	0.4602	1.03 (0.97, 1.08)	0.3719
Diastolic blood pressure (mmHg)	≥80	‐	‐	‐	‐	‐	‐	‐	‐	ref	ref	ref	ref
	<80	‐	‐	‐	‐	‐	‐	‐	‐	1.6 (0.7, 2.6)	0.0007	0.90 (0.86, 0.95)	<0.0001
Microalbuminuria	Yes	‐	‐	‐	‐	‐	‐	‐	‐	ref	ref	ref	ref
	No	‐	‐	‐	‐	‐	‐	‐	‐	0.2 (−1.0, 1.4)	0.7543	0.96 (0.90, 1.02)	0.2217
Macroalbuminuria	Yes	‐	‐	‐	‐	‐	‐	‐	‐	ref	ref	ref	ref
	No	‐	‐	‐	‐	‐	‐	‐	‐	−1.7 (−4.1, 0.7)	0.1634	1.04 (0.92, 1.19)	0.5172
Cancer diagnosis	Yes	‐	‐	‐	‐	‐	‐	‐	‐	ref	ref	ref	ref
	No	‐	‐	‐	‐	‐	‐	‐	‐	1.7 (−0.4, 3.9)	0.1124	0.90 (0.80, 1.00)	0.0517
Physical activity^b^	< once per week	‐	‐	‐	‐	‐	‐	‐	‐	ref	ref	ref	ref
	2‐3 times/week	‐	‐	‐	‐	‐	‐	‐	‐	0.0 (−1.3, 1.4)	0.9436	1.00 (0.93, 1.07)	0.9838
	4‐5 times a week	‐	‐	‐	‐	‐	‐	‐	‐	1.8 (0.4, 3.1)	0.0087	0.92 0.86, 0.98)	0.0154
	Daily	‐	‐	‐	‐	‐	‐	‐	‐	1.1 (−0.2, 2.3)	0.0952	0.96 (0.90, 1.02)	0.1832
Smoker	Yes^c^	‐	‐	‐	‐	‐	‐	‐	‐	ref	ref	ref	ref
	No	‐	‐	‐	‐	‐	‐	‐	‐	4.3 (3.0, 5.5)	<0.0001	0.82 (0.78, 0.88)	<0.0001

a If aged ≥65 years and unemployed.

b 30‐min walk or equivalent.

c At least 1 cigarette/pipe per day or stopped smoking within 3 months.

### 3.3 Persistence

In the total study population, 72.5% of patients were persistent for at least 1 year and 56.3% were persistent for 3 years (Table 2). Mean persistence was 758 days. Nearly 8% of all patients filled only 1 prescription for lipid‐lowering medicines. Among patients who discontinued treatment, 25% did so within the first year. The discontinuation rate decreased to 5% during the third year and was on average higher among patients without previous CVD. In patients with previous CVD, censoring resulting from filled prescriptions for multi‐dose dispensed medicines and death was 9.2% and 8.4%, respectively, compared with 2.1% and 2.4%, respectively, in patients without previous CVD.

**Figure 2:**
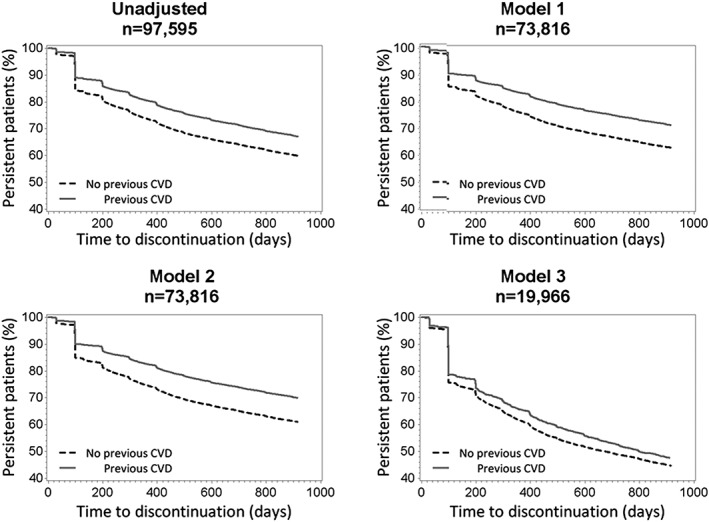
Kaplan‐Meier persistence curves for persistence to lipid‐lowering medicines in patients with type 2 diabetes by previous CVD. Model 1 adjusted for socioeconomic status; model 2 adjusted for socioeconomic status and concurrent medicines; and model 3 adjusted for socioeconomic status, concurrent medicines, and clinical characteristics

Adjusted for potential confounders (Table 3), hazard ratios for discontinuation by previous CVD was 0.75–0.91 (*P*<0.0001), indicating a lower risk for discontinuation compared with patients without previous CVD. The fully adjusted model showed that patients born in Africa and the Americas had a 60% and 80% increased risk for discontinuation, respectively, compared with patients born in Sweden (*P* < 0.0001). Risk for discontinuation was 20% lower in non‐smokers and patients who used other glucose‐lowering medicines than insulin compared with smokers and patients who did not fill any prescriptions for diabetes medicines (the references) (*P* < 0.0001). Even when considering potential confounders, Kaplan‐Meier persistence curves showed greater treatment persistence among patients with previous CVD compared with patients without previous CVD (Figure 2).

### 3.4 Sensitivity analysis

When the gap length cutoff alternated between 30, 45, and 90 days, the proportion of non‐adherent patients in the total population was 67.2%, 61.1%, and 49.7%, respectively. Independent of the cutoff, the proportion of non‐adherent patients was 10% higher among patients without previous CVD compared with patients with previous CVD (*P* < 0.0001). When we set the discontinuation gap length cutoff at 180 days for non‐censored patients and alternated between 90, 135, and 180 days for patients who were censored during the observation period, the proportion of patients in the total population who discontinued treatment was 36.6%, 36.2%, and 35.8%, respectively. Depending on alternated cutoff, the change in 1‐year and 2‐year persistence was ≤0.4%. We observed no difference in 3‐year persistence.

## 4 DISCUSSION

This nation‐wide register‐based study assessed refill adherence and persistence to lipid‐lowering medicines in 97 595 patients with type 2 diabetes. To our knowledge, our study is the largest cohort for studying adherence and persistence to lipid‐lowering medicines restricted to patients with type 2 diabetes. Moreover, our results offer a unique opportunity to consider clinical characteristics. Due to our 3‐year observation period, differences in results compared with other studies (with 1 or 2 years of observation) might be due to an association between longer observation and increased risk for discontinuation.^11^

The mean 3‐year MPR for lipid‐lowering medicines reported here was 71% among patients with type 2 diabetes; 55% had an MPR above 80%. This is consistent with previous studies that report 66%–87% in mean 12‐month MPR to lipid‐lowering medicines (mean value = 74%) among patients with diabetes and/or previous CVD; 51% of the patients had an MPR measure >80%.^11^, ^13^ Thus, patients in our study showed greater adherence to lipid‐lowering treatment considering the longer observation period. The maximum gap method showed that approximately 40% of our patients were adherent compared with 65% of statin users in the general population of Sweden during a 2‐year observation period.^35^ This is possibly due to differences in study population and observation period, but also because patients who filled only 1 statin prescription were excluded in the previous study, generating higher estimated refill adherence. Approximately 8% of our total study population filled only 1 prescription for lipid‐lowering medicines.

Almost 75% of our patients were persistent for least 1 year and the discontinuation rate decreased over time. Our results concur with a previous study in the general population of Finland, which reported that 69% of its patients were persistent to statin treatment for at least 1 year using the 180‐day discontinuation gap cutoff.^36^ The majority of Finnish patients discontinued treatment within the first year, and 44% of the population remained persistent after 10 years of follow‐up. Altogether, these findings suggest that the first year with lipid‐lowering medicines is crucial for the continuation of treatment, providing valuable information for health care providers to consider when treating patients with moderate to high CVD risk.

Additionally, patients who filled prescriptions for other glucose‐lowering and/or antihypertensive medicines had a higher refill adherence and longer persistence to lipid‐lowering medicines. This is consistent with an earlier study that showed higher adherence to statins with increasing number of concurrent medicines (to a certain threshold).^37^ That finding is positive because the treatment approach for diabetes may involve multiple medicines. Furthermore, patients born in Africa or the Americas were significantly less adherent and more likely to discontinue treatment. Although these patients represent only 2% of the total study population, the difference in adherence and persistence compared with patients born in Sweden could be due to misunderstandings and language difficulties between patient and health care provider^38^, ^39^ that will require early discussion between patient and provider to facilitate adequate use of medicines, including sufficient adherence to treatment.

### 4.1 Strengths and limitations

The most important strengths of this study are its national coverage of patients and its use of data from national registers. Such data provide a great advantage for studying refill adherence instead of prescription adherence that lacks information about whether prescriptions are filled. However, we cannot assure that our patients actually ingested the filled medicines. Another limitation is the reduction of patients included in the fully adjusted model. NDR coverage of patients was 50%‐80% in 2007–2010, thus limiting the availability of data on clinical characteristics because not all covariates were measured annually. Nevertheless, our fully adjusted model included nearly 20 000 patients.

Altogether 9 out of 10 users of lipid‐lowering medicines with type 2 diabetes were included in the final study population after applying the exclusion criteria. The study population was limited to patients with type 2 diabetes who filled prescriptions for lipid‐lowering medicines in monotherapy. Thus, patients using combination of lipid‐lowering medication were excluded, corresponding to 3.7% of all new users of lipid‐lowering medicines with type 2 diabetes. Combination of lipid‐lowering substances is recommended only in patients with several risk factors of CVD and who do not reach target valued for LDL‐cholesterol with monotherapy. We also excluded patients with multi‐dose dispensed medicines which may limit the generalizability of our findings somewhat in the oldest part of the population as the majority of multi‐dose dispensed medicines is issued to patients ≥65 years of age. Thus, our study population might be younger and have less comorbidity than the general population of type 2 diabetes patients in Sweden.

## 5 CONCLUSIONS

In this nation‐wide study of patients with type 2 diabetes, refill adherence to lipid‐lowering medicines was 71%. Almost 75% of patients were persistent for 1 year, and 56% were persistent 3 years. Patients with previous CVD showed higher refill adherence and longer persistence compared with patients without previous CVD. This information is valuable and important for consideration by health care providers who treat patients with type 2 diabetes and moderate to high risk for CVD.

## CONFLICT OF INTEREST STATEMENT

Karolina Andersson Sundell is employed by AstraZeneca. However, the views expressed in this study are her own and not those of AstraZeneca. The remaining authors declare no conflict of interest.

## Supporting information


**Appendix 1.** Classification of cardiovascular disease and cancer.
