# Supplementary material for: Refill adherence and persistence to lipid‐lowering medicines in patients with type 2 diabetes: A nation‐wide register‐based study
Source: Pharmacoepidemiol Drug Saf. 2017 Aug 11;26(10):1220–32. doi: 10.1002/pds.4281 (PMC5656892; doi:10.1002/pds.4281)
Supplement: Supplementary file 1 — Appendix 1. Classification of cardiovascular disease and cancer. [file PDS-26-1220-s001.pdf]

Appendix 1. Classification of cardiovascular disease and cancer.

Complications	ICD-10 codes	Operation codes
Ischemic heart disease	I20-I25	
Atrial fibrillation	I48	
Heart failure	I50	
Cerebrovascular disease	I61, I63, I64, I67.9	
Peripheral vascular disease /leg amputation	I70.2, I73.1, I73.9, I79.2, E10.5, E11.5, E14.5	NHQ09, NHQ11, NGQ09, NGQ11, NGQ99, NFQ09, NFQ19, NFQ99, NEQ19, NEQ99
Coronary artery bypass graft		FNA00, FNA10, FNA20, FNA96, FNB00, FNB20, FNB96, FNC10, FNC20, FNC30, FNC40, FNC50, FNC60, FNC96, FND10, FND20, FND96, FNE00, FNE10, FNE20, FNE96, FNF00, FNF10, FNF20, FNF30, FNF96
Percutaneous coronary intervention		FNG00, FNG02, FNG05, FNG06, FNG10, FNG30, FNG96
Cancer	C00-C97	
